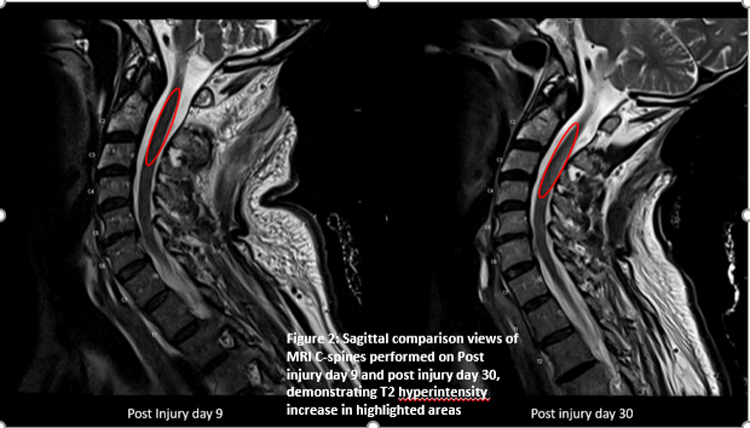# 566 Posterior Spinal Cord Syndrome after High Voltage Electrical Injury

**DOI:** 10.1093/jbcr/irad045.162

**Published:** 2023-05-15

**Authors:** Ariel Rodgers, Callie Thompson, Daniel Murphy, Irma Fleming, Giavonni Lewis, Christopher LaChapelle, Greg Hobson

**Affiliations:** University of Utah, Salt Lake City, Utah; University of Utah, Salt Lake City, Utah; University of Utah, Salt Lake City, Utah; University of Utah Burn Center, Salt Lake City, Utah; University of Utah Health, Salt Lake City, Utah; University of Utah, Salt Lake City, Utah; University of Utah, Salt Lake City, Utah; University of Utah, Salt Lake City, Utah; University of Utah, Salt Lake City, Utah; University of Utah, Salt Lake City, Utah; University of Utah Burn Center, Salt Lake City, Utah; University of Utah Health, Salt Lake City, Utah; University of Utah, Salt Lake City, Utah; University of Utah, Salt Lake City, Utah; University of Utah, Salt Lake City, Utah; University of Utah, Salt Lake City, Utah; University of Utah, Salt Lake City, Utah; University of Utah Burn Center, Salt Lake City, Utah; University of Utah Health, Salt Lake City, Utah; University of Utah, Salt Lake City, Utah; University of Utah, Salt Lake City, Utah; University of Utah, Salt Lake City, Utah; University of Utah, Salt Lake City, Utah; University of Utah, Salt Lake City, Utah; University of Utah Burn Center, Salt Lake City, Utah; University of Utah Health, Salt Lake City, Utah; University of Utah, Salt Lake City, Utah; University of Utah, Salt Lake City, Utah; University of Utah, Salt Lake City, Utah; University of Utah, Salt Lake City, Utah; University of Utah, Salt Lake City, Utah; University of Utah Burn Center, Salt Lake City, Utah; University of Utah Health, Salt Lake City, Utah; University of Utah, Salt Lake City, Utah; University of Utah, Salt Lake City, Utah; University of Utah, Salt Lake City, Utah; University of Utah, Salt Lake City, Utah; University of Utah, Salt Lake City, Utah; University of Utah Burn Center, Salt Lake City, Utah; University of Utah Health, Salt Lake City, Utah; University of Utah, Salt Lake City, Utah; University of Utah, Salt Lake City, Utah

## Abstract

**Introduction:**

High voltage electrical injuries have been called “the grand masquerader”, and significant neurological sequalae have been described. Here, we report the case of a 73-year-old man who sustained a 14.5% total body surface area (TBSA) full thickness electrical burns, most significantly to his scalp (Figure 1). On initial evaluation, there was concern for loss of proprioception resulting in gait instability. A magnetic resonance image (MRI) of the cervical spine performed on post injury day 9 showed no evidence of cervical spinal cord injury.

**Methods:**

A novel descriptive case report of a high-voltage electrical injury with incomplete spinal cord injury

**Results:**

The patient underwent several operative interventions for wound coverage and preservation of function with the known challenges experienced with high voltage burn wounds. Despite lack of imaging confirmation, suspicion for an occult neurological injury remained high. Neurological consultation confirmed limited proprioception and loss of 2-point discrimination. Due to these specific findings that resulted in an inability to make significant rehabilitation gains, a subsequent MRI of his cervical spine performed on post-injury day 30 demonstrated T2 hyperintensity in the dorsal column in the cervical spine at the C2-3 and C5-6 levels, suggestive of myelopathy (Figure 2).

**Conclusions:**

To our knowledge, this is the first reported case of an incomplete spinal cord injury (posterior spinal cord syndrome in this case) due to an electrical injury without bony abnormality the association of paralysis. With the knowledge of this injury, our burn therapists have been able to develop a rehabilitation plan with reasonable expectation and goals. While discussing prognosis with the patient and his family, we noted the absence of data regarding outcomes after injuries of this nature and sought to contribute to the literature with this case.

**Applicability of Research to Practice:**

A novel case of delayed imaging confirmation of posterior cord syndrome contributes to the body of evidence for neurological sequelae due to electrical injuries.